# Cardiac biomarkers and health-related quality of life in new hemodialysis patients without symptomatic cardiac disease

**DOI:** 10.1186/2054-3581-1-16

**Published:** 2014-07-15

**Authors:** Christopher E Williams, Bryan M Curtis, Edward W Randell, Robert N Foley, Patrick S Parfrey

**Affiliations:** Division of Nephrology, Health Sciences Center, Memorial University, St. John’s, Newfoundland A1B 3 V6 Canada; Laboratory Medicine, Faculty of Medicine, Memorial University, St. John’s, Newfoundland Canada; Chronic Disease Research Group, University of Minnesota, Minneapolis, MA USA

## Abstract

**Background:**

Health Related Quality of Life (HRQOL) is impaired in hemodialysis patients and cardiac biomarkers are elevated, but their relationship is uncertain.

**Objectives:**

To determine whether the cardiac biomarkers, troponin T and N terminal pro-B type natriuretic peptide (NT-proBNP), predict deterioration in the physical domains of HRQOL.

**Design:**

A prospective cohort study of patients in a randomized controlled clinical trial of correction of anemia with erythropoietin.

**Setting:**

Multiple hemodialysis centers located throughout Canada and Europe.

**Participants:**

Patients who started maintenance hemodialysis within the previous 3–18 months, with no clinical evidence or prior history of symptomatic cardiac failure or ischemic heart disease, and left ventricular volume < 100 ml/m^2^.

**Measurements:**

*Predictor*: Baseline concentrations of Troponin T and NT-proBNP. *Outcomes*: Physical function and vitality scores using the SF-36 questionnaire and fatigue scores using the FACIT questionnaire at baseline and after 24, 48, and 96 weeks follow-up.

**Methods:**

Univariate analysis of the association between baseline variables and baseline HRQOL scores and change in scores over time was undertaken using linear regression. Multivariate models were created using multiple linear regression, and it was pre-specified that these include the variables which were associated with the outcome at a p < 0.05 in the univariate regression.

**Results:**

Baseline median (interquartile range) physical function score was 70 (50–85), vitality 55 (40–75), and fatigue 73 (58–86). The 75th percentile for Troponin T was 0.05 ng/mL and for NT-proBNP 652 ng/mL. High Troponin T levels were significantly associated with deterioration in the 3 physical domains, independent of other risk factors, whereas high NT-proBNP were not associated. In multivariate models baseline Troponin T > 0.05 ng/mL were significantly associated with the change from baseline to 96 weeks follow-up for SF-36 vitality and FACIT-fatigue scores, and approached statistical significance for SF-36 physical function (0.056).

**Limitations:**

Not possible to confirm whether Troponin T associations were independent of subsequent cardiac events.

**Conclusions:**

In hemodialysis patients without prior symptomatic cardiac disease and without a dilated left ventricle at baseline, elevated baseline Troponin T levels, but not NT-pro BNP, were independently associated with deterioration in the physical domains of HRQOL.

**Electronic supplementary material:**

The online version of this article (doi:10.1186/2054-3581-1-16) contains supplementary material, which is available to authorized users.

## Abrégé

**Contexte:**

Chez les patients en hémodialyse, la qualité de vie liée à la santé (QVLS) est diminuée, et les biomarqueurs cardiaques sont élevés, mais la relation entre les deux est incertaine.

**Objectifs:**

Déterminer si les biomarquers cardiaques, la troponine T et le N-terminal peptide natriurétique de type B (NT-ProBNP), prédisent la détérioration des domaines physiques de la QVLS.

**Type d’étude:**

Une étude prospective de cohorte sur des patients dans le cadre d’un essai clinique contrôlé randomisé concernant la correction de l’anémie à l’aide de l’érythropoïétine.

**Contexte:**

Plusieurs centres d’hémodialyse situés au Canada et en Europe, coordonnés de façon centralisée à partir de St. John’s, au Canada, pour les patients canadiens, et de Manchester, en Angleterre, pour les patients européens.

**Participants:**

Les patients en hémodialyse chronique de plusieurs centres, qui ont entamé une dialyse au cours des derniers 3 à 18 mois, et qui ne présentent aucune donnée clinique et aucun antécédent d’insuffisance cardiaque symptomatique ou de cardiopathie ischémique, mais un volume ventriculaire gauche < 100 ml/m^2^.

**Mesures:**

*Prédicteur*: concentration de référence de Troponine T et de NT-proBNP. *Résultats:* Degré de vitalité et fonction physique à l’aide du questionnaire SF-36 et degré de fatigue à l’aide du questionnaire FACIT, au point de référence, puis après le suivi des 24, 48 et 96 semaines.

**Méthodes:**

L’analyse unidimensionnelle de l’association entre les variables fondamentales et les scores de référence de la QVLS, et la variation dans les scores a été entreprise au moyen de la régression linéaire. Les modèles multivariables ont été créés en utilisant la régression linéaire multiple, et il a été pré-spécifié que ceux-ci comprenaient les variables associées aux résultats de p < 0,05 dans la régression à une variable.

**Résultats:**

Le score médian de référence (écart interquartile) de la fonction physique était de 70 (50–85), celui de la vitalité de 55 (40–75), et celui de la fatigue 73 (58–86). Le 75^e^ percentile de la troponine T était de 0,05 ng/mL, et pour le NT-proBNP, de 652 ng/mL. Les niveaux élevés de troponine T étaient associés de façon significative à la détérioration des 3 domaines physiques, indépendants des autres facteurs de risque, alors que les niveaux élevés de NT-proBNP ne démontraient aucune association significative. Dans les modèles multivariables, un niveau fondamental de troponine T > 0,05 ng/mL était associé de façon significative à la variation entre le point de référence et le suivi des 96 semaines en ce qui a trait aux scores SF-36 de vitalité et FACIT de fatigue, et avaient une certaine signification statistique à une fonction physique de SF-36 (0,056).

**Limites de l’étude:**

Impossible de confirmer si les associations avec la troponine T étaient indépendantes d’événements cardiaques subséquents.

**Conclusions:**

Chez les patients en hémodialyse ne présentant pas d’antécédents de maladie cardiaque symptomatique ni une dilatation du ventricule gauche au point de référence, des niveaux élevés de troponine T au point de référence, mais pas de NT-proBNP, ont été indépendamment associés à une détérioration des domaines physiques de QVLS.

**Trial registration:**

Clinical trials.gov # NCT00261521.

## What was known before

Patients with ESRD have diminished scores in the physical domains of health related quality of life. Biomarkers for adequacy of dialysis, mineral metabolism and inflammation correlate poorly with these domains, although nutritional biomarkers do correlate.

## What the study adds

In hemodialysis patients without prior symptomatic cardiac disease elevated baseline Troponin T levels, but not B type natriuretic peptide, are associated with deterioration in the physical domains of quality of life.

## Background

Health Related Quality of Life (HRQOL) is a consistent predictor of mortality in end stage renal disease (ESRD) ( [1–3]). Patients with ESRD have consistently diminished HRQOL compared with matched controls without ESRD, most pronounced in physical function and vitality ( [4]). Biomarkers are regularly tracked in dialysis units, but biomarkers for adequacy of dialysis (KT/V), mineral metabolism and inflammation correlate poorly with HRQOL, although nutritional biomarkers associate with the physical domains in HRQOL ( [4]). Although HRQOL scores are substantially higher in hemodialysis (HD) patients without prior symptomatic cardiac disease compared to unselected hemodialysis patients ( [5, 6]) the relationship between cardiac biomarkers, such as N terminal pro-B type naturetic peptide (NT-proBNP) and troponin T, and HRQOL is uncertain.

Cardiovascular events, particularly heart failure and atherosclerotic events have a major impact on HRQOL in ESRD ( [7]). Hemodialysis patients who have not experienced these events may have elevated troponin T, a marker of myocardial injury, or elevated NT-proBNP, a marker of left ventricular (LV) wall stress ( [8]). To determine whether these cardiac biomarkers associate with poor HRQOL or predict deterioration in HRQOL we analyzed troponin T and NT-proBNP levels in incident HD patients, without prior cardiac failure or ischemic heart disease events and without overt cardiac dilation (N = 596), followed for 2 years in a multinational, blinded, randomized controlled trial ( [8, 9]), in whom HRQOL was measured at baseline and serially during follow-up using the SF-36 and FACIT fatigue questionnaires ( [5]).

We hypothesized that cardiac biomarkers would predict deterioration in the physical domains of HRQOL, independent of other baseline clinical characteristics. The objectives of this report are to quantify the associations between traditional biomarkers and cardiac biomarkers, measured at baseline in incident HD patients without symptomatic cardiac disease, and baseline measures of physical function, vitality, and fatigue, together with short-term (24 weeks) and long-term (1 and 2 years) changes in these domains.

## Methods

The cohort studied in this paper was enrolled in a randomized trial of correction of anemia with erythropoietin ( [9]). The design, methods, randomization, and epoetin dosing protocols have been reported previously ( [9]). Patients were randomly assigned to one of the following hemoglobin (Hb) targets: 9.5 to 11.5 (low target) or 13.5 to 14.5 g/dl (high target). Patients were masked to treatment assignment, as were their doctors. Mean Hb levels at the end of the initial 24-week titration phase were 13.3 and 10.9 g/dl, respectively. During the maintenance phase, from weeks 24 through 96, corresponding mean Hb levels were 13.1 and 10.8 g/dl. Cardiac structure constituted the primary study outcome, and no difference was observed between the two groups ( [9]). Consequently, for the purpose of this article, all patients were included as one cohort. The study was centrally coordinated from St. John’s, Canada, for Canadian patients and Manchester, England, For European patients.

Local independent ethics committees or institutional review boards approved the study protocol form before study initiation. The study was conducted in accordance with Good Clinical Practice guidelines and the Declaration of Helsinki. All patients gave informed consent before study enrollment.

### Study population

Inclusion criteria were as follows: Age ≥ 18 years; inception of maintenance HD within the previous 3 to 18 months; predialysis Hb level between 8 and 12 g/dl; LV volume index (LVVI) < 100 ml/m^2^, and predialysis diastolic BP (DBP) < 100 mmHg. Exclusion criteria were as follows: Clinical evidence or history of symptomatic cardiac failure or ischemic heart disease; daily prednisone dosages ≥ 10 mg; medical conditions that are likely to reduce epoetin responsiveness, including uncorrected iron deficiency; concurrent malignancy; blood transfusion in the preceding month; therapy with cytotoxic agents; seizure in the preceding year; hypersensitivity to intravenous iron; and current pregnancy or breastfeeding.

### Description of study procedures

Laboratory tests were measured centrally by Quest Diagnostics (Van Nuys, CA, and Heston, UK). With the high target, the treatment goal was increments of 0.5 to 1.0 g/dl every 2 weeks, until achieving stability between 13.5 and 14.5 g/dl. Other treatment goals included predialysis diastolic blood pressure between 70 and 90 mmHg, urea reduction ratio > 67%, and transferrin saturation ≥ 20%. On a weekly basis, midweek predialysis blood pressure levels were communicated weekly to and treatment recommendations sent from the coordinating center. These were determined by the protocols in use at the individual treating centers, and a single, formal, standardized method was not imposed. The last patient completed the study in May 2003.

Quality of life was assessed using the KDQOL and FACIT fatigue questionnaires ( [10, 11]). We pre-specified the domains of HRQOL for this analysis: physical function and vitality measured using the SF-36 questionnaire and fatigue using the FACIT fatigue questionnaire. Higher scores reflect better health. These were also the pre-specified domains for HRQOL outcomes in the trial ( [5]). A-priori we defined short-term change in HRQOL as the change from baseline to the assessment at 24 weeks, and long-term change as the change from baseline to the assessments made at 48 and 96 weeks.

There was no difference in the clinical or demographic characteristics or in treatment assignment of those who had only one KDQOL assessment n = ( [12]) and those who had serial assessments (n = 484). At each assessment, > 90% of patients remaining in the study completed the questionnaire.

### Sample size estimate

The sample size needed to detect a 15% difference between treatment groups in the primary outcome (left ventricular cavity volume index) was calculated as 166 per treatment group, given a two-tailed significance of 0.05, a power of 0.90, and an SD of the percentage change in left ventricular cavity volume index of 42% ( [9]). With an expected dropout rate of 40%, primarily as a result of transplantation, 277 patients were required for each treatment group.

At baseline SF-36 was completed in 457 patients and FACIT fatigue in 572 patients. The comparable numbers at 24 weeks were 443 and 499, at 48 weeks 435 and 432, and at 96 weeks 335 and 325.

### Risk factors

In addition to demographic factors (age, gender, and race), baseline clinical characteristics (diabetes, primary renal disease, dialysis vintage, blood pressure, body mass index, type of vascular access) were recorded, as were conventional laboratory tests results (Hb, white cell count, percentage of iron saturation, urea reduction ratio, and serum albumin) and other serologic tests at baseline. Serum concentrations of N terminal pro-B type natriuretic peptide (NT-proBNP) and cardiac troponin T were measured in 2009 using diagnostic kits and performed on an Elecsys 2010 immunochemistry analyzer (Roche Diagnostics, Montreal, Quebec, Canada). Serum high-sensitivity C-reactive protein was measured using CRP reagent (Beckman Coulter, Fullerton, CA) and performed on an IMMAGE Immunochemistry system (Beckman Coulter). Serum IL-6 was measured using IL-6 kits and performed on a Unicel DxI 800 Access Immunoassay system (Beckman Coulter). Serum leptin was measured by ELISA technique using kits purchased from Diagnostic Systems Laboratories, Inc (Webster, Tx).

### Statistical analysis

Baseline characteristics were described by number (%) or by median with interquartile range. For the cardiac and inflammatory biomarkers high levels were pre-specified as above the 75th percentile. Univariate analysis of the association between baseline variables and baseline HRQOL scores and change in scores over time was undertaken using linear regression. Multivariate models were created using multiple linear regression, and it was pre-specified that these include the variables which were associated with the outcome at a p < 0.05 in the univariate regression. In addition multivariate models were created to determine the effect of troponin T levels on change in HRQOL independent of age, sex, diabetes and baseline HRQOL score.

## Results

**Figure 1:**
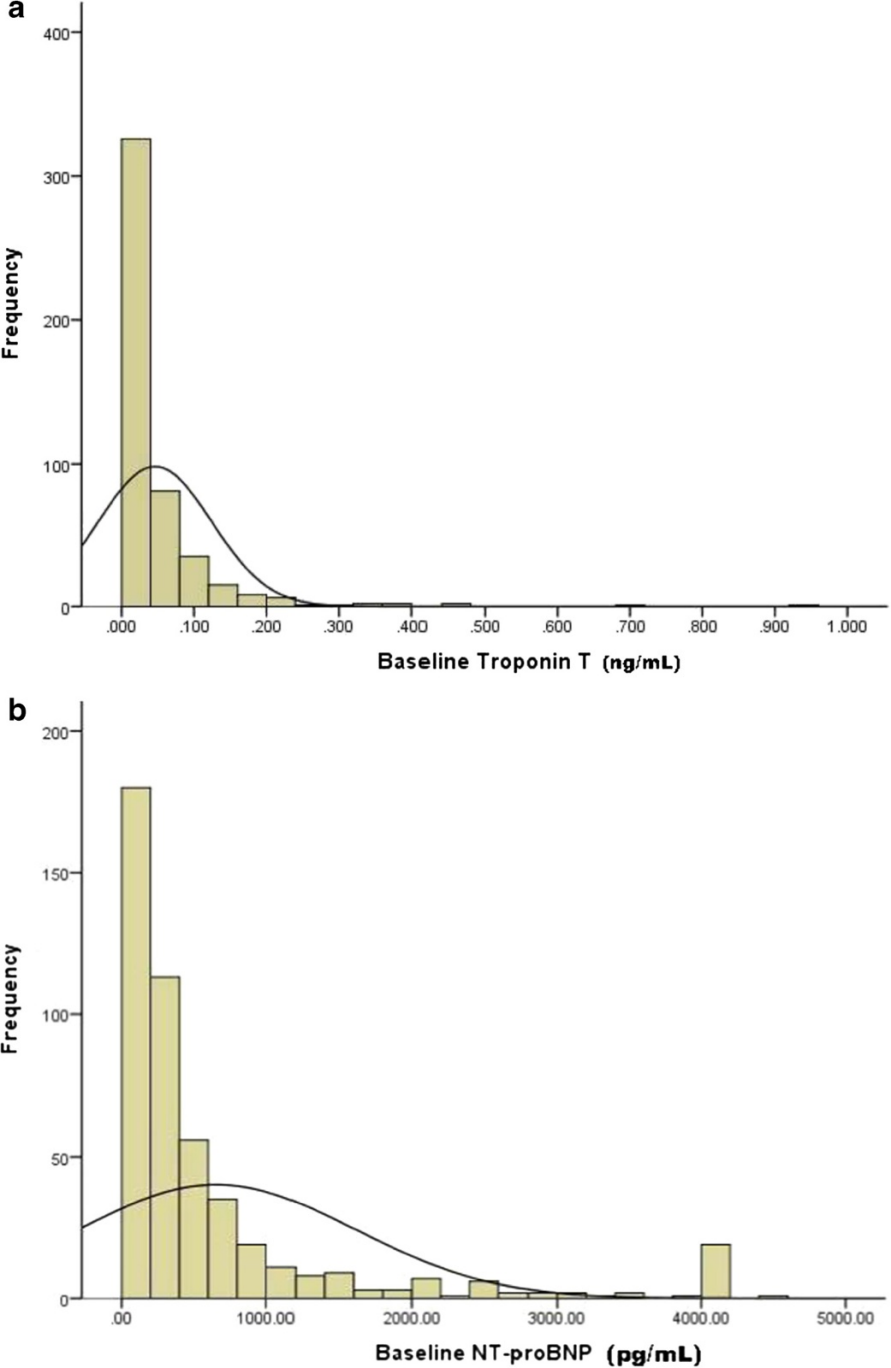
**Distribution of baseline troponin T levels (A) and NT-proBNP (B) levels in hemodialysis patients without prior symptomatic cardiac disease.** Troponin T levels are expressed in units of ng/mL and NT-proBNP in units of pg/mL).

A total of 596 patients were enrolled in 95 treatment centers in 10 countries between February 2000 and June 2001. Table 1 shows baseline characteristics. Median age was 51.5 years, 39.6% were female, and the vast majority were white. Only 17.8% had diabetes as the cause of ESRD, and patients with a clinical history of cardiac failure and ischemic heart disease were excluded. Incident patients who started dialysis between 3 and 18 months before study entry were enrolled, and median time on dialysis was 9 months. The vast majority (84.2%) had a fistula as vascular access. The distribution of troponin T and for NT-proBNP at baseline is displayed in Figure 1 A and B. The upper quartile for troponin T was > 0.051 ng/ml (N = 118), and for NT-proBNP it was > 652 pg/ml (N = 120).

**Table 1:** **Baseline demographic and clinical characteristics, biomarkers and HRQOL scores**

Age (yrs); median (25th to 75th percentiles)	51.5 (39 to 62)
Female; N (%)	236 (39.6)
Non-white race; N (%)	63 (10.6)
Cause of ESRD:	
Glomerulonephritis; N (%)	171 (28.7)
Diabetic Nephropathy; N (%)	106 (17.8)
Polycistic kidney disease; N (%)	54 (9.1)
Hypertension; N (%)	48 (8.1)
Other/Unknown; N (%)	217 (36.4)
Dialysis duration (mo); median (25th to 75th percentiles)	9 (6 to 14)
Dialysis access:	
Fistula; N (%)	502 (84.2)
Graft; N (%)	33 (5.5)
Catheter; N (%)	61 (10.2)
Assigned to high Hb target; N (%)	296 (49.7)
Epoetin dosage (U/wk); median (25th to 75th percentiles); n = 584	6000 (4000 to 8000)
BMI (kg/m^2^); median (25th to 75th percentiles)	25.5 (22.6 to 29.3)
Systolic BP (mmHg); median (25th to 75th percentiles); n = 595	140 (130 to 158)
Diastolic BP (mmHg); median (25th to 75th percentiles); n = 596	80 (71 to 90)
Hemoglobin (g/dl); median (25th to 75th percentiles); n = 580	11 (10.2 to 11.7)
Serum albumin (g/L); median (25th to 75th percentiles); n = 588	40 (38 to 41)
Urea reduction ratio (%); median (25th to 75th percentiles); n = 572	67 (60 to 72.5)
Biomarkers; median (25th to 75th percentiles)	
Cardiac: Troponin T (ng/ml); n = 481	0.021 (0.009 to 0.051)
NT-proBNP (pg/ml); n = 481	289.2 (137.3 to 651.9)
Inflammatory: C-Reactive protein (mg/L); n = 481	3.47 (1.47 to 8.13)
IL-6 (pg/ml); n = 481	4.39 (2.69 to 8.8)
Leptin (ng/ml); n = 481	12.4 (3.7 to 43.8)
Quality of life domains; median (25th to 75th percentiles)	
SF-36 Physical functioning; n = 457	70 (50 to 85)
SF-36 Vitality; n = 457	55 (40 to 75)
FACIT Fatigue; n = 572	73.1 (57.7 to 86.3)

### Serial HRQOL scores

**Figure 2:**
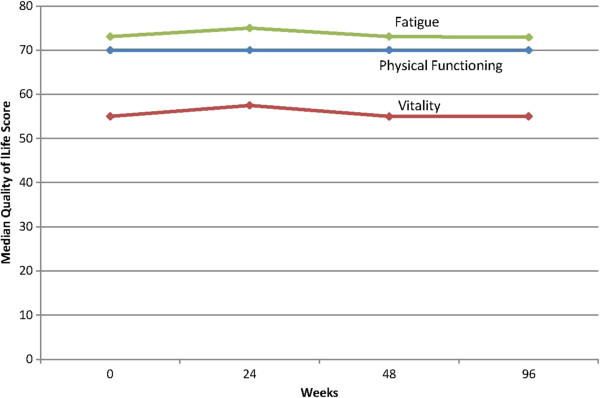
**Serial HRQOL scores for SF-36 domains physical function and vitality, and for FACIT fatigue, in hemodialysis patients without symptomatic cardiac disease.**

Median score for SF-36 physical function was 70, for SF-36 vitality it was 55, and for FACIT fatigue 73 (Table 1). Figure 2 shows the median scores at baseline, 24, 48, and 96 weeks for the cohort.

### Associations with baseline HRQOL scores

Table 2 shows the significant univariate associations between baseline clinical variables and biomarkers with HRQOL scores. Multivariate models revealed that the significant, independent variables associated with better SF-36 physical function were younger age, presence of a fistula, and lower NT-proBNP. For higher SF-36 vitality and FACIT fatigue scores only higher URR was identified as a significant predictor (Table 2). Additional file 1: Table S1 shows the results of analysis of the other SF-36 domains, which were not pre-specified.

**Table 2:** **Significant associations between baseline clinical variables and biomarkers and baseline HRQOL scores, using univariate and multivariate analysis**

Characteristic	Reference	Unadjusted B coefficient	95% C.I.	Adjusted B coefficient	95% C.I.
**SF-36 Physical functioning**					
Female	Male	-7.56	-12.30 to -2.81**		
Age	Per 1 year	-0.49	-0.63 to -0.35**	-0.38	-0.56 to -0.20**
Body Mass Index	Per 1 kg/m^2^	-0.63	-1.05 to -0.20**		
Diabetes	No Diabetes	-8.25	-14.27 to -2.23**		
Fistula	No Fistula	12.52	6.54 to 18.51**	10.69	3.52 to 17.86**
Serum Albumin	≤ 40 g/L	6.36	1.54 to 11.18**		
Serum Creatinine	Per 1 umol/L	0.02	0.01 to 0.03**		
Sodium	Per 1 mmol/L	0.76	0.06 to 1.46*		
White Blood Cells	≤ 8.1 × 10^9^/L	-7.98	-13.60 to -2.37**		
Neutrophils	≤ 68%	-7.33	-12.90 to -1.77**		
Leptin	Per 1 ng/ml	-0.17	-0.26 to -0.08**		
Troponin T	≤ 0.05 ng/ml	-9.54	-15.60 to -3.48**		
NT-proBNP	≤ 651.9 pg/ml	-6.05	-12.11 to 0.01*	-6.33	-12.40 to -0.26*
IL-6	Per 1 pg/ml	-0.19	-0.38 to -0.01*		
**SF-36 Vitality**					
Female	Male	-5.73	-9.88 to -1.58**		
Urea Reduction Ratio	≤ 60%	8.58	3.52 to 13.64**	6.33	0.48 to 12.17*
White Blood Cells	≤ 8.1 × 10^9^/L	-6.76	-11.62 to -1.91**		
Leptin	Per 1 ng/ml	-0.09	-1.17 to -0.01*		
C-reactive protein	Per 1 mg/L	-0.18	-0.35 to -0.00*		
**FACIT Fatigue**					
Female	Male	-5.00	-8.26 to -1.73**		
Body Mass Index	Per 1 kg/m^2^	-0.30	-0.60 to 0.00*		
Diabetes	No Diabetes	-5.07	-9.40 to -0.73*		
Urea Reduction Rate	≤ 60%	6.75	3.04 to 10.47**	5.61	1.42 to 9.81**
Leptin	Per 1 ng/ml	-0.10	-0.17 to -0.04**		

*p ≤ 0.05; **p ≤ 0.01.

Unadjusted B coefficient was calculated using univariate linear regression (column 3 and 4).

Adjusted B coefficient was calculated using multiple linear regression to identify the significant and independent predictors (column 5 and 6).

### Associations with change in HRQOL

Table 3 shows the significant univariate and multivariate associations for both short-term (24 weeks) and long-term (48 and 96 weeks) change in HRQOL for the 3 domains of interest. High troponin T levels at baseline were significantly and independently associated with decrease in SF-36 physical function at 48 weeks, decrease in SF-36 vitality at 96 weeks and decrease in FACIT fatigue at 24 weeks, independent of other variables significantly associated with the outcome. In a second multivariate model high troponin T levels were significantly associated with deterioration in physical function at 24, 48, and 96 weeks, decrease in vitality at 96 weeks, and decrease in fatigue at 24 weeks, independent of age, sex, diabetes and baseline HRQOL score (Table 4). Examination of the unadjusted and adjusted B coefficients (Table 4) suggest consistent impact of baseline troponin T levels on change in HRQOL. General linear models for repeated measures demonstrated that high troponin T levels were significantly associated with decrease in physical function scores (p < 0.001), decrease in vitality of borderline significance (p = 0.06), decrease in fatigue scores (p = 0.04).

**Table 3:** **Significant associations between baseline clinical variables and biomarkers and change in short-term and long-term HRQOL scores, using univariate and multivariate analysis**

Characteristic	Reference	Unadjusted B coefficient	95% C.I.	Adjusted B coefficient	95% C.I.
**SF-36 Vitality**					
Change at 24 wks					
High Hb Group	Low Hb Group	5.09	1.16 to 9.03*	5.11	1.07 to 9.14*
Platelet Count	≤ 260 × 10^9^/L	-5.00	-9.63 to -0.38*	-4.99	-9.58 to -0.40*
Change at 48 wks					
Urea reduction ratio	≤ 60%	-6.97	-12.22 to -1.72**	-7.06	-12.35 to 1.77**
Serum calcium	≤ 2.42 mmol/L	5.34	0.94 to 9.73*	6.18	1.62 to 10.74**
Platelet count	≤ 260 × 10^9^/L	-5.93	-10.67 to -1.18*	-5.01	-9.84 to -0.18*
Epo dose	≤ 6000 U/wk	5.25	1.07 to 9.43*	5.73	1.53 to 9.93**
Change at 96 wks					
Fractional shortening	Per 1%	-0.32	-0.62 to -0.02*		
Troponin T	≤ 0.051 ng/ml	-7.55	-14.18 to -0.91*	-7.09	-13.73 to -0.46*
**SF-36 Physical functioning**					
Change at 24 wks					
Left Ventricular Mass Index	Per 1 g/m^2^	-0.06	-0.12 to -0.00*		
Serum Calcium	≤ 2.42 mmol/L	4.25	0.02 to 8.47*		
Platelet Count	≤ 260 × 10^9^/L	-5.54	-9.95 to -1/13	-5.93	-10.37 to -1.50**
Change at 48 wks					
Serum Calcium	≤ 2.42 mmol/L	6.63	1.61 to 11.66**	6.00	0.39 to 11.62*
Troponin T	≤ 0.051 ng/ml	-6.40	-12.62 to -0.18*	-6.70	-12.93 to -0.47*
Change at 96 wks					
Diabetes	No Diabetes	-7.28	-14.07 to -0.49*	-8.84	-16.61 to -1.07*
Troponin T	≤ 0.051 ng/ml	-9.43	-16.44 to -2.42**		
**FACIT Fatigue**					
Change at 24 wks					
Lactase dehydrogenase	Per 1 U/L	-0.05	-0.09 to -0.00*		
White blood cells	≤ 8.1 × 10^9^/L	-4.32	-8.19 to -0.46*		
Troponin T	≤ 0.051 ng/ml	-5.38	-9.37 to -1.38**	-4.91	-9.09 to -0.74*
Change at 48 wks					
Serum creatinine	Per 1 umol/L	0.01	0.00 to 0.02**	0.01	0.00 to 0.02*
Platelet Count	≤ 260 × 10^9^/L	-5.06	-9.20 to -0.93*	-4.78	-8.92 to -0.63*
Change at 96 wks					
Epo Dose	≤ 6000 U/wk	-5.11	-9.92 to -0.31*		

*p ≤ 0.05; **p ≤ 0.01.

Unadjusted B coefficient was calculated using univariate linear regression (column 3 and 4).

Adjusted B coefficient was calculated using multiple linear regression to identify the significant and independent predictors (column 5 and 6).

**Table 4:** **The unadjusted and adjusted B coefficients for baseline Troponin T levels and baseline HRQOL scores and change in these scores over time, using univariate and multivariate analysis**

	Unadjusted B	p value	Adjusted B*	p value*	Adjusted B**	p value**
**SF-36 Physical functioning**						
Baseline	-9.54	0.002	-5.92	0.071		
Change at 24 wks	-4.13	0.111			-6.02	0.018
Change at 48 wks	-6.40	0.044	-6.70	0.035	-8.09	0.008
Change at 96 wks	-9.43	0.009	-7.60	0.056	-8.42	0.015
**SF-36 Vitality**						
Baseline	-1.56	0.571				
Change at 24 wks	-3.46	0.182			-3.23	NS
Change at 48 wks	-1.36	0.633			-1.36	NS
Change at 96 wks	-7.55	0.026	-7.09	0.036	-7.10	0.037
**FACIT Fatigue**						
Baseline	-1.58	0.465				
Change at 24 wks	-5.38	0.008	-4.91	0.021	-5.95	0.002
Change at 48 wks	-3.89	0.093			-4.22	NS
Change at 96 wks	-5.46	0.057	-6.19	0.035	-4.56	NS

*Adjusted for other variables with p ≤ 0.05.

**Adjusted for age, sex, diabetes status and baseline QoL score.

Unadjusted B coefficient was calculated using univariate linear regression.

Adjusted B coefficient was calculated using multivariate linear regression.

Baseline NT-proBNP levels were not predictive of change in HRQOL (Additional file 1: Table S2), nor were biomarkers of inflammation (CRP and ILS) or leptin. Additional file 1: Table S3 shows the results from analysis of the other SF-36 domains, which were not pre-specified.

### Clinical associations with baseline Troponin T

Table 5 shows the significant univariate and multivariate associations between high troponin T levels at baseline and baseline clinical characteristics and biomarkers. The independent and significant predictors of high levels were male sex, older age, diabetes, high lactic dehydrogenase and high NT-proBNP levels.

**Table 5:** **Significant associations between baseline Troponin T levels and baseline clinical characteristics, using univariate and multivariate analysis**

Characteristic	Reference	Unadjusted B coefficient	95% C.I.	Adjusted B coefficient	95% C.I.
Sex	Male	0.52	0.33 to 0.82**	0.38	0.22 to 0.67***
Age	Per 1 year	1.03	1.01 to 1.04**	1.02	1.00 to 1.04*
Diabetes	No Diabetes	4.59	2.81 to 7.48***	4.62	2.55 to 8.38***
LV Mass Index	1 g/m^2^	1.01	1.01 to 1.02***		
Hb	≤ 11.1 g/dL	0.52	0.33 to 0.80**		
Serum Albumin	≤ 40 g/L	0.56	0.36 to 0.88*		
Lactate Dehydrogenase	1 U/L	1.015	1.01 to 1.02***	1.01	1.00 to 1.02*
Potassium	1 mmol/L	1.36	1.03 to 1.79*		
White Blood Cells	≤ 8.1 × 10^9^/L	1.84	1.15 to 2.95*		
Platelet Count	≤ 260 × 10^9^/L	1.73	1.09 to 2.75*		
NT-proBNP	≤ 651.9 pg/ml	4.00	2.55 to 6.28***	3.53	2.04 to 6.13***

*p ≤ 0.05; **p ≤ 0.01; ***p ≤ 0.001.

Unadjusted B coefficient was calculated using univariate linear regression (column 3 and 4).

Adjusted B coefficient was calculated using multivariate linear regression to identify the significant and independent predictors (column 5 and 6).

## Discussion

The major conclusions in this paper were that in new hemodialysis patients without symptomatic cardiac disease:

1. HRQOL scores for physical function, vitality and fatigue were closer to those for the general population than for unselected hemodialysis patients, and on average changed little over 2 years of follow-up.
2. troponin T levels were elevated at baseline in a substantial minority of patients, and high values were significantly associated with high NT-proBNP levels independent of age, sex and diabetes.
3. high troponin T levels at baseline were significantly associated with decrease in physical function, vitality and fatigue, whereas NT pro-BNP levels were not.

The mean score in the general population for SF-36 physical function was 86 and for dialysis patients 41, ( [12, 6]), whereas in our patients without symptomatic cardiac disease the score was 67 ( [5]). For SF-36 vitality the comparable scores were 66, 43 and 58, and for FACIT fatigue the mean score in the general population was 80, in diabetic CKD patients 30 ( [13]) and in our group 70. Clearly the presence of symptomatic cardiac disease is associated with HRQOL in dialysis patients. The current paper demonstrates that in dialysis patients without symptomatic cardiac disease at baseline few biomarkers were consistently associated with the deterioration in quality of life scores, but cardiac damage revealed by high Troponin T levels was associated with deterioration. It is important to note that the associations between change in HRQOL scores and high troponin T levels were not confounded by the prior occurrence of symptomatic heart failure or ischemic heart disease, or by the presence of LV dysfunction defined by a high LV volume. The fact that this group was relatively healthy is confirmed by the observation of an annual mortality of 5%, substantially lower than that in unselected hemodialysis patients ( [9]).

In the study by Apple et al. ( [14]) using the 99th percentile of a healthy reference population to define an elevated troponin T level, 82% of unselected asymptomatic hemodialysis patients had a level above this value ( > 0.01 ng/ml). In our study the interquartile range was 0.01 to 0.05 ng/ml. It is likely that elevated troponin T levels in dialysis patients reflects not only myocardial injury and ischemia but also myocardial fibrosis ( [15]). A meta-analysis suggested that an elevated troponin T > 0.1 ng/ml can identify a subgroup of dialysis patients with poor survival and higher risk of death ( [16]). In a previous paper we reported that higher troponin T levels were predictive of subsequent cardiovascular events or death in our selected cohort, but that this association was not independent of age, diabetes, systolic blood pressure or NT-proBNP levels ( [8]). The current paper lends support to the belief that high troponin levels are not innocuous, and that they are associated with deterioration in the physical domains of HRQOL. Whether this effect is mediated via the development of subsequent cardiac events which cause a decrease in HRQOL or by the degree of myocardial injury present at baseline is difficult to determine. The fact that high Troponin T levels were associated with lower HRQOL scores at baseline and after short-term follow-up suggests that the latter hypothesis is tenable, particularly as only 4% of cases developed a cardiovascular/death event by six months (myocardial infarction, myocardial ischemia, angina pectoris, cardiac failure, pulmonary edema, cerebellar infarction, cerebral hemorrhage, cerebral vascular disorder, or death) ( [8]). Whether this interpretation is correct requires further investigation.

BNP or NT-proBNP is a cardiac biomarker reflecting LV wall stress that may occur secondary to volume expansion, pressure overload and increased wall tension ( [15]). In our previous paper NT-proBNP in this selected group was an independent predictor of baseline LVMI, of increase in LVMI over time, and of the subsequent occurrence of cardiovascular events or death ( [8]). Although higher BNP levels may be associated with left ventricular hypertrophy and with systolic dysfunction, in stable hemodialysis patients with normal LV function on echocardiography high BNP levels are likely the result of blood volume expansion and decrease with reduction in post dialysis “dry” weight ( [17]). In the current paper, high BNP levels were not associated with a decrease HRQOL in the short-term or the long-term.

Limitations of the study include: 1. the study from which the data originated was not designed to evaluate the impact of cardiac biomarkers on HRQOL. 2. It was not possible to confirm that baseline Troponin T levels were predictive of HRQOL independent of subsequent cardiac events. However the group studied limited confounding induced by the presence of symptomatic cardiac disease and of echocardiographic systolic dysfunction. 3. It is possible that patients who did not have repeat measurements of HRQOL may be systematically different but this would likely lead to underestimate of the true association between the biomarkers and HRQOL.

## Conclusion

We conclude that in a group of patients without prior symptomatic cardiac disease at baseline or without a dilated LV, elevated troponin T levels, but not elevated BNP levels, on starting hemodialysis are associated with deterioration in the physical domains of HRQOL.

## Electronic supplementary material

Additional file 1: Table S1: Significant associations between baseline clinical variables and biomarkers and six baseline SF-36 HRQOL domains not pre-specified. **Table S2.** The unadjusted and adjusted B coefficients for baseline NT pro-BNP levels and baseline HRQOL scores and changes in these scores over time. **Table S3.** Significant associations between baseline clinical variables and biomarkers for change in six baseline SF-36 HRQOL domains not pre-specified, using univariate and multivariate analysis. (DOCX 21 KB)
