# Supplementary material for: Cardiac biomarkers and health-related quality of life in new hemodialysis patients without symptomatic cardiac disease
Source: Can J Kidney Health Dis. 2014 Jul 15;1:16. doi: 10.1186/2054-3581-1-16 (PMC4452142; doi:10.1186/2054-3581-1-16)
Supplement: Supplementary file 1 — Additional file 1: Table S1: Significant associations between baseline clinical variables and biomarkers and six baseline SF-36 HRQOL domains not pre-specified. Table S2. The unadjusted and adjusted B coefficients for baseline NT pro-BNP levels and baseline HRQOL scores and changes in these scores over time. Table S3. Significant associations between baseline clinical variables and biomarkers for change in six baseline SF-36 HRQOL domains not pre-specified, using univariate and multivariate analysis. (DOCX 21 KB) [file 40697_2014_15_MOESM1_ESM.docx]

**Supplementary Table 1: Significant associations between baseline clinical variables and biomarkers and six baseline SF-36 HRQOL domains not pre-specified.**

| **Characteristic** | **Reference** | **Unadjusted B Coefficient** | **95% C.I.** | **Adjusted B Coefficient** | **95% CI** |
| --- | --- | --- | --- | --- | --- |
| **SF-36 Role Physical** | | | | | |
| Urea Reduction Ratio | ≤ 60% | 14.25 | 4.59 to 23.91** |  |  |
| Adjusted Calcium | ≤ 2.42 mmol/L | -9.83 | -18.25 to -1.41* | -15.95 | -25.61 to -6.30** |
| Lactase Dehydrogenase | Per 1 U/L | -0.13 | -0.24 to -0.02* | -0.18 | -0.33 to -0.03* |
| White Blood Count | ≤ 8.1 x 10^9^/L | -10.18 | -19.66 to -0.70* |  |  |
| Troponin T | ≤ 0.051 ng/ml | -13.43 | -23.72 to -3.14* |  |  |
| NT-proBNP | ≤ 651.9 pg/ml | -10.39 | -20.64 to -0.14* |  |  |
|  |  |  |  |  |  |
| **SF-36 Mental Health** |  |  |  |  |  |
| High Hb Group | Low Hb Group | -6.13 | -9.83 to -2.43** | -5.63 | -9.89 to -1.38** |
| Female | Male | -5.69 | -9.52 to -1.86** |  |  |
| Urea Reduction Ratio | ≤ 60% | 8.73 | 4.10 to 13.36** | 8.63 | 3.33 to 13.92** |
| Creatinine | per 1 umol/L | 0.01 | 0.00 to 0.02* |  |  |
| Lactase Dehydrogenase | per 1 U/L | -0.06 | -0.12 to -0.01* | -0.09 | -0.16 to -0.02** |
| Sodium | per 1 mmol/L | 0.70 | 0.14 to 1.26* |  |  |
| White Blood Count | ≤ 8.1 x 10^9^/L | -5.08 | -9.59 to -0.57* |  |  |
| Leptin | Per 1 ng/ml | -0.08 | -0.16 to -0.01* |  |  |
|  |  |  |  |  |  |
| **SF-Role Emotional** |  |  |  |  |  |
| Urea Reduction Rate | ≤ 60% | 17.58 | 8.17 to 26.99** | 16.28 | 6.85 to 25.70** |
| Sodium | Per 1 mmol/L | 1.55 | 0.43 to 2.67** | 1.34 | 0.22 to 2.47* |
|  |  |  |  |  |  |
| **SF-36 Social Function** |  |  |  |  |  |
| Urea Reduction Rate | ≤ 60% | 6.39 | 0.75 to 12.04* | 6.04 | 0.43 to 11.65* |
| Lactase Dehydrogenase | Per 1 U/L | -0.09 | -0.16 to -0.03** | -0.09 | -0.16 to -0.03** |
|  |  |  |  |  |  |
| **SF-36 Bodily Pain** |  |  |  |  |  |
| Fistula | No Fistula | 10.10 | 3.71 to 16.50** | 10.09 | 2.17 to 18.02* |
| LV Mass Index | per 1 g/m^2^ | -0.08 | -0.15 to –0.01* |  |  |
| Lactase Dehydrogenase | per 1 U/L | -0.08 | -0.15 to -0.00* | -0.10 | -0.19 to 0.02* |
| Leptin | per 1 ng/ml | -0.14 | -0.24 to -0.04** | -0.13 | -0.23 to -0.02* |
|  |  |  |  |  |  |
| **SF-36 General Health** |  |  |  |  |  |
| Age | per 1 year | 0.14 | 0.02 to 0.27* |  |  |
| Urea Reduction Rate | ≤ 60% | 8.70 | 3.72 to 13.68** | 7.39 | 1.52 to 13.26* |
| hsCRP | per 1 mg/L | -0.21 | -0.39 to -0.04* | -0.22 | -0.39 to -0.05* |
|  |  |  |  |  |  |
| ***p ≤ 0.05; **p ≤ 0.01** |  |  |  |  |  |

Unadjusted B coefficient was calculated using univariate linear regression (column 3 and 4).

Adjusted B coefficient was calculated using multiple linear regression to identify the significant and independent predictors (column 5 and 6).

**Supplementary Table 2: The unadjusted and adjusted B coefficients for baseline NT pro-BNP levels and baseline HRQOL scores and changes in these scores over time**

| **Physical Functioning** | **Unadjusted B**  **Coefficient** | **p value** | **Adjusted B**  **Coefficient** | **p value** |
| --- | --- | --- | --- | --- |
| Baseline | -6.05 | 0.05 | -6.33 | 0.041 |
| Change at 24 wks | 2.09 | 0.429 |  |  |
| Change at 48 wks | 0.57 | 0.864 |  |  |
| Change at 96 wks | 5.18 | 0.164 |  |  |
|  |  |  |  |  |
| **Vitality** |  |  |  |  |
| Baseline | -1.28 | 0.641 |  |  |
| Change at 24 wks | 0.64 | 0.810 |  |  |
| Change at 48 wks | 4.20 | 0.157 |  |  |
| Change at 96 wks | -4.08 | 0.241 |  |  |
|  |  |  |  |  |
| **Fatigue** |  |  |  |  |
| Baseline | -2.13 | 0.318 |  |  |
| Change at 24 wks | -0.76 | 0.713 |  |  |
| Change at 48 wks | -1.96 | 0.409 |  |  |
| Change at 96 wks | -3.44 | 0.242 |  |  |

**Supplementary Table 3: Significant associations between baseline clinical variables and biomarkers for change in six baseline SF-36 HRQOL domains not pre-specified, using univariate and multivariate analysis**

| **Characteristic** | **Reference** | **Unadjusted B Coefficient** | | **95% C.I.** | **Adjusted B Coefficient** | **95% CI** |
| --- | --- | --- | --- | --- | --- | --- |
| **SF-36 Role Physical** | | | | | | |
|  | | | | | | |
| Change at 24 wks | | | | | | |
| Serum Calcium | ≤ 2.42 mmol/L | 12.51 | | 3.46 to 21.56** | 11.27 | 2.02 to 20.51* |
| Neutrophils | ≤ 68% | -12.34 | | -22.25 to -2.43* | -12.06 | -21.89 to -2.23 |
| Change at 48 wks | | | | | | |
| Serum Albumin | ≤ 40 g/L | 10.63 | | -0.01 to 21.26* |  |  |
| Serum Calcium | ≤ 2.42 mmol/L | 13.90 | | 3.11 to 24.69* | 13.40 | 2.64 to 24.17* |
| Change at 96 wks | | | | | | |
| No predictive Factors |  |  | |  |  |  |
|  |  |  | |  |  |  |
| **SF-36 Mental Health** |  |  | |  |  |  |
|  | | | | | | |
| Change at 24 wks | | | | | | |
| Serum Calcium | ≤ 2.42 mmol/L | 3.92 | | 0.02 to 7.81* | 6.14 | 1.74 to 10.54** |
| Platelet Count | ≤ 260 x 10^9^/L | -4.31 | | -8.42 to -0.20* | -5.60 | -10.19 to -1.01* |
| hsCRP | per 1 mg/L | 0.19 | | 0.04 to 0.34* | 0.20 | 0.06 to 0.35** |
| Change at 48 wks | | | | | | |
| Fistula | No Fistula | 8.47 | | 3.42 to 13.52** |  |  |
| Change at 96 wks | | | | | | |
| Serum Creatinine | per 1 umol/L | -0.01 | | -0.02 to -0.00* |  |  |
|  |  |  | |  |  |  |
| **SF-36 Role Emotional** |  |  | |  |  |  |
|  | | | | | | |
| Change at 24 wks | | | | | | |
| Lactase Dehydrogenase | Per 1 U/L | -0.14 | | -0.26 to -0.02* |  |  |
| Change at 48 wks |  |  | |  |  |  |
| Fistula | No Fistula | 23.36 | | 10.76 to 35.96** | 20.22 | 7.25 to 33.19** |
| Serum Albumin | ≤ 40 g/L | 11.15 | | 0.96 to 21.34* |  |  |
| White Blood Count | ≤ 8.1 x 10^9/^/L | -14.52 | | -26.32 to -2.72* | -13.11 | -24.77 to -1.45* |
| Change at 96 wks |  |  | |  |  |  |
| Body Mass Index | Per 1 kg/m^2^ | -1.18 | | -2.17 to -0.19* |  |  |
| White Blood Count | ≤ 8.1 x 10^9^/L | -13.54 | | -26.81 to -0.28* |  |  |
|  |  |  | |  |  |  |
| **SF-36 Social Function** |  |  | |  |  |  |
|  |  |  | |  |  |  |
| Change at 24 wks |  |  | |  |  |  |
| White Blood Count | ≤ 8.1 x 10^9^/L | -7.81 | | -13.68 to -1.94** | -6.73 | -12.87 to -0.59* |
| Platelet Count | ≤ 260 x 10^9^/L | -5.61 | | -11.14 to -0.08* |  |  |
| Change at 48 wks |  |  | |  |  |  |
| Serum Calcium | ≤ 2.42 mmol?l | 7.34 | | 1.63 to 13.04* |  |  |
| Change at 96 wks |  |  | |  |  |  |
| No Predictive Factors |  |  | |  |  |  |
| **SF-36 Bodily Pain** |  |  | |  |  |  |
|  |  |  | |  |  |  |
| Change at 24 wks |  |  | |  |  |  |
| No Predictive Factors |  |  | |  |  |  |
| Change at 48 wks |  |  | |  |  |  |
| Diabetes | No Diabetes | 8.22 | | 0.83 to 15.61* |  |  |
| White Blood Count | ≤ 8.1 x 10^9^/L | -9.16 | | -15.86 - -2.46** |  |  |
| Change at 96 wks |  | |  |  |  |  |
| Hemoglobin | ≤ 11.1 g/dL | | -6.69 | -13.34 to -0.03* |  |  |
| Epo Dosage | ≤ 6000 units/wk | | -7.46 | -14.25 to -0.66* |  |  |
| Leptin | Per 1 ng/ml | | -0.14 | -0.27 to -0.02* | -0.13 | -0.27 to 0.00* |
|  |  | |  |  |  |  |
| **SF-36 General Health** |  | |  |  |  |  |
| Change at 24 wks |  | |  |  |  |  |
| No Predictive Factors |  | |  |  |  |  |
| Change at 48 wks |  | |  |  |  |  |
| Urea Reduction Ratio | ≤ 60% | | -5.71 | -10.87 to -0.56* |  |  |
| Change at 96 wks |  | |  |  |  |  |
| Age | Per 1 year | | -0.18 | -0.33 to -0.04* |  |  |
| Hemoglobin | ≤ 11.1 g/dL | | -5.92 | -10.67 to -1.18* |  |  |
| Transferrin Saturation | ≤ 32% | | 5.19 | 0.40 to 9.97* |  |  |
| Leptin | per 1 ng/ml | | -0.10 | -0.19 to -0.01* |  |  |
